# New Drugs, Appliances, and Things Medical

**Published:** 1893-04-01

**Authors:** 


### NEW DRUGS, APPLIANCES, AND THINGS MEDICAL.

[All preparations, appliances, novelties, etc., of which a notice is desired, should be sent for the Editor, to care of The Manager, 428, Strand, London, W.C.]

### MILLED TOILET SOAPS.

(Messrs. Phillips, Hoskins, and Co., Frome Bride Soap Works, Bristol.)

We have received samples of Messrs. Phillips and Hoskins' Cleopatra toilet soaps, milled in the French manner. They are excellent in quality, and possess marked cleansing properties. The manner of their preparation gives firmness to their texture, and this, added to their low price, causes these soaps to be most economical. The specimens submitted to us, Windsor, Rose, and Honey, are all very highly scented; but as the scent is very evanescent, and is practically non-existent after a few days' use, this quality should not interfere with the popularity which these soaps should enjoy beyond that of those which are more delicately perfumed and less well made. The cleansing nature of Messrs. Phillips and Hoskins' soaps should especially commend those prepared for household use.

